# Meta-analysis of association between CT-based features and tumor spread through air spaces in lung adenocarcinoma

**DOI:** 10.1186/s13019-020-01287-9

**Published:** 2020-09-10

**Authors:** Qifan Yin, Huien Wang, Hongshang Cui, Wenhao Wang, Guang Yang, Peng Qie, Xuejiao Xun, Shaohui Han, Huining Liu

**Affiliations:** 1 Department of Thoracic Surgery, Hebei Provincal General Hospital, 348, West He-Ping Road, Shijiazhuang, 050051 Hebei Province People’s Republic of China; 2 Department of Pharmacy, Hebei Provincal General Hospital, 348, West He-Ping Road, Shijiazhuang, 050051 Hebei Province People’s Republic of China

**Keywords:** Spread through air spaces, CT-based features, Lung adenocarcinoma, Association, Meta-analysis

## Abstract

**Objective:**

Spread through air space (STAS) is a novel invasive pattern of lung adenocarcinoma and is also a risk factor for recurrence and worse prognosis of lung adenocarcinoma after sublobar resection. The aims of this study are to evaluate the association between computed tomography (CT)-based features and STAS for preoperative prediction of STAS in lung adenocarcinoma, eventually, which could help us choose appropriate surgical type.

**Methods:**

Systematic research was conducted to search for studies published before September 1, 2019. The association between CT-based features of radiological tumor size>2 cm、pure solid nodule、 part-solid nodule or Percentage of solid component (PSC)>50% and STAS was evaluated. According to rigorous inclusion and exclusion criteria. Eight studies including 2385 patients published between 2015 and 2018 were finally enrolled in our meta-analysis.

**Results:**

Our results clearly depicted that there is no significant relationship between radiological tumor size>2 cm and STAS with the combined OR of 1.47(95% CI:0.86–2.51). Meta-analysis of 3 studies showed that pure solid nodule in CT image were more likely to spread through air spaces with pooled OR of 3.10(95%CI2.17–4.43). Meta-analysis of 5 studies revealed the part-solid nodule in CT image may be more likely to appear STAS in adenocarcinoma (ADC) (combined OR:3.10,95%CI:2.17–4.43). PSC>50% in CT image was a significant independent predictor in the diagnosis of STAS in ADC from our meta-analysis with combined OR of 2.95(95%CI:1.88–4.63).

**Conclusion:**

In conclusion, The CT-based features of pure solid nodule、part-solid nodule、PSC>50% are promising imaging **biomarkers** for predicting STAS in ADC and may substantially influence the choice of surgical type. In future, more studies with well-designed and large-scale are needed to confirm the conclusion.

## Introduction

With the wide use of low-dose helical computed tomography (LDCT) and high resolution computed tomography (HRCT) screening in lung cancer, the number of patients with early stage lung cancer characterized as pulmonary nodule has been found to be increasing before they become a unresectable lesion. However, lung cancer remains the first cancer-related death in both men and women [1]. Beyond infiltration of myofibroblast stroma and lymph vascular and pleural invasion, spread through air space (STAS) is regarded as a novel invasion pattern of lung adenocarcinoma, even though there are some controversies [2, 3]. The conception of STAS was first introduced into our vision by Kadota and colleagues in 2015 [4]. In the 2015 World Health Organization (WHO) Classification [5], STAS was newly recognized as a pattern of tumor spread in lung adenocarcinoma. STAS is defined as micropapillary clusters, solid nests, or single cells spreading within air spaces beyond the edge of the main tumor [6]. STAS can be found in 14.8 to 56.4% of lung adenocarcinomas and has been proven to be a risk factor for survival and reoccurrence after operation [4, 7–10]. Comparing with STAS-negative tumors, lung adenocarcinomas with STAS positive showed a significant worse recurrence-free survival and overall survival [10]. However, if surgical operation type was considered, sublobar resection of STAS-positive tumors has been reported to be associated with a high risk of distant and locoregional recurrence, while such association was not observed in patients undergoing lobectomy [4]. Therefore, preoperative knowledge of the presence of STAS may facilitate appropriate surgery type choosing.

As we all know, lobectomy and systematic lymph nodes dissection is the standard operation for the early stage lung cancer patients. In recent years, several studies indicated similar survival between sublobar resection and lobectomy for stage IA NSCLC [11–14], Compared with those who underwent traditional lobectomy. Patients who underwent sublobectomy had less lung tissue resected and more lung function preserved, The sublobar resection surgical approaches included wedge resection and segmentectomy. However, small adenocarcinoma with STAS-positive should be treated by lobectomy combined with systematic dissection lymph nodes, not sublobar resection. If we can predict the STAS through the CT-based features before surgical resection, that would aid us in the selection of the optimum surgical procedure. The purpose of our meta-analysis is to evaluate the association between CT-based features and STAS and help us to predict STAS before surgery, eventually, which could help us choose appropriate surgical type.

## Methods

### Literature search strategy

We performed a systematic literature search through the following databases without date limitation: PubMed, Cochrane Library, Ovid and Web of Science databases. The search was updated to September 1, 2019. The main search terms included: “STAS” (e.g., “spread through air space”, and “spread through air spaces”,) and “lung cancer”[e.g., “lung neoplasm”, “lung carcinoma”,“non-small cell lung cancer (NSCLC)”, “small cell lung cancer (SCLC)”] and “CT-based features”(e.g.,“CT features”, “Computed Tomography features”, “CT manifestations”, “CT characteristics”). The reference list was also checked for relevant articles.

### Inclusion and exclusion criteria

The eligible studies were evaluated by two authors based on the inclusion criteria as follows: (1) studied patients with ADC were pathological examination confirmed; (2) STAS was confirmed by pathological examination; (3) studied patients underwent primary curative surgical resection; (4) correlation of STAS with CT features or CT manifestations was reported;(5) a single nodule. Articles didn’t meet inclusion criteria would be excluded. Exclusion criteria were as follows: (1) abstracts, letters, case reports, reviews or nonclinical studies; (2) patients who underwent neoadjuvant chemotherapy and those with incomplete resection; (3) multiple nodules;(4) studies were not written in English; (5) patients were not lung adenocarcinoma.

### Data extraction and quality assessment

The following data were extracted by two independent investigators; first author, publication year, nation, number of participants, participants characteristics (age, gender, the state of STAS, stage, CT-based features) and odd ratio (OR) and their corresponding 95% confidence interval (95%CI). Articles that could not be categorized based on title and abstract alone were retrieved for full-text review. If disagreement occurred, two investigators discussed and reached consensus with a third investigator. Given all studies are retrospective observational studies, The Newcastle-Ottawa Scale (NOS) was used to assess each of the included studies quality by two independent authors. The NOS consists of three parts: selection (0-4points), comparability (0–2 points), and outcome assessment (0–3 points). NOS scores of≥6 were regarded as high-quality studies.

### Statistical analysis

We pooled OR corresponding 95% CI to assess the association between CT-based features and STAS in lung ADC. The heterogeneity amid studies was tested by I^2^ statistic; If test results showed I^2^ value > 50%, we considered high heterogeneity within studies and the random effect model was applied; If not (I^2^ value≤50%), the fixed effect model was used. Additionally, if there is considerable heterogeneity, meta regression analysis with restricted maximum likelihood (REML) method and subgroup analysis will be conducted. Funnel plot and Egger’s test were used to estimate publication bias. To evaluate the stability of the results, we conducted sensitivity analysis to test it. These statistic data was performed using STATA version 12.0. A *p* < 0.05 was considered statistically significant differences.

## Results

### Search results

**Fig. 1:**
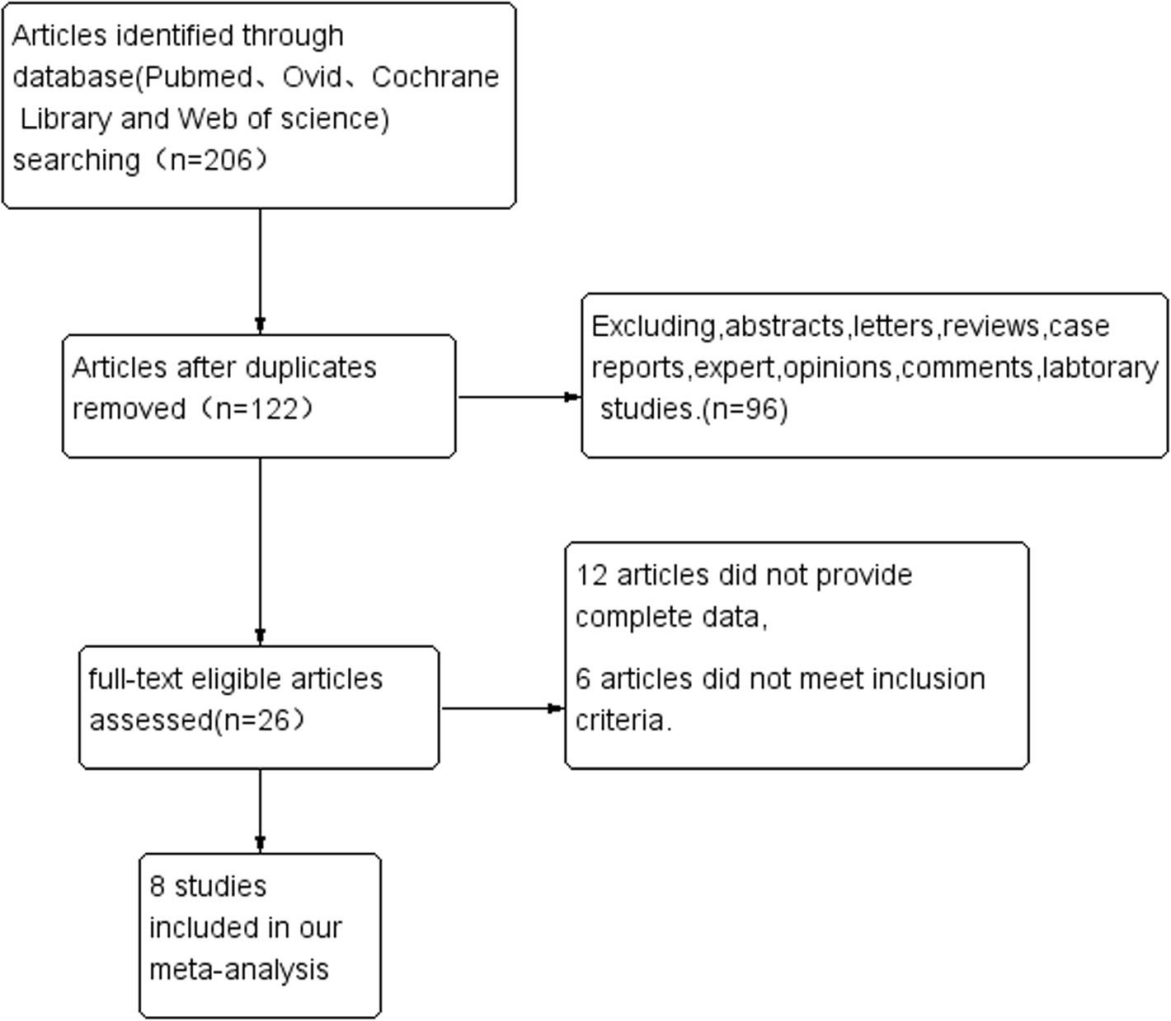
The flow diagram for retrieving eligible articles

A total of 206 articles were searched:120 from Pubmed, 24 from Ovid,20 from Cochrane Library and 42 from Web of science. After carefully inspection of these articles, 8 studies including 2385 patients published between 2015 and 2018 were finally enrolled in our meta-analysis. The detail processes of study selection were showed in the flow diagram (Fig. 1). Among them, five studies were from Japan, one study was performed in USA, China and Republic of Korea, respectively. OR and 95%CI were extracted directly or calculated indirectly in 8 studies. In our meta analysis, not every association has been evaluated using every paper included in the meta-analysis; four studies revealed the association between radiological tumor size>2 cm and STAS, three studies illuminated the relation between pure solid nodule and STAS, five studies depicted the connection between part-solid nodule and STAS, two studies demonstrated the association between percentage of solid component (PSC)>50% and STAS. The PSC was calculated as follows: [maximum diameter of the solid component/maximum diameter of the lesion] × 100, where the maximum diameter of the lesion includes both ground-glass opacity and solid component in CT image. The characteristics of the enrolled studies were shown in Table 1.

**Table 1:** The basic characteristics of enrolled studies

author	year	region	N(M/F)	year	STAS(+/−)	subtype	stage	Lobectomy/sublobar Resection	NOS	CT-based features
Kadota et al. [4]	2015	Japan	411 (164/247)	68	155/256	ADC	I	291/120	7	part-solid nodule.
Shiono et al. [15]	2016	Japan	318 (149/169)	70	47/271	ADC	I	202/116	5	pure solid nodule.
Dai et al. [10]	2017	China	383 (178/205)	60	116/267	ADC	I	364/19	6	radiological tumor size>2 cm.
Masai et al. [16]	2017	Japan	508 (248/260)	66	76/432	ADC	I	0/508	7	part-solid nodule.
Toyokawa et al. [17]	2018	Japan	327 (153/174)	69	191/136	ADC	I-IV	235/84	7	radiological tumor size>2 cm,pure solid nodule,part-solid nodule.
de Margerie-Mellon et al. [18]	2018	USA	80 (27/53)	68	40/40	ADC	NA	NA	6	radiological tumor size>2 cm, part-solid nodule.
Kim et al. [19]	2018	Republic of Korea	276 (129/147)	59	92/184	ADC	I-III	226/50	6	pure solid nodule, part-solid nodule.
Toyokawa et al. [20]	2018	Japan	82 (40/42)	71	31/51	ADC	I	0/82	7	radiological tumor size>2.0 cm.

### The association between radiological tumor size>2 cm and STAS in ADC

**Fig. 2:**
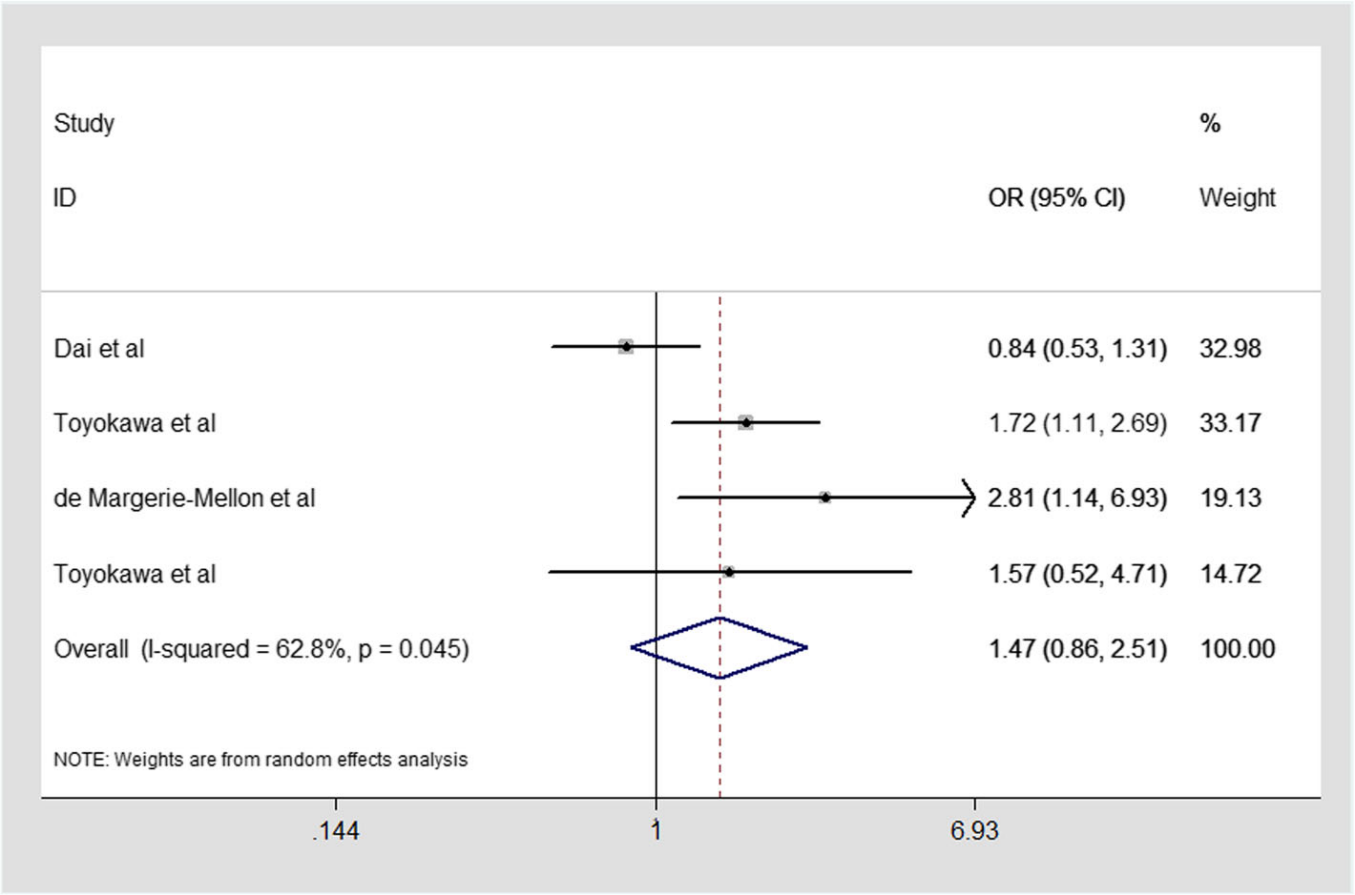
Meta-analysis of the association between radiological tumor size>2 cm and STAS in ADC

The results of the association are showed in Fig. 2. Four studies presented the data to evaluated the association between radiological tumor size>2 cm and STAS in our meta-analysis. Considering the heterogeneity (I^2^ = 62.8%, *P* = 0.045). Therefore, a random-effect model was applied. Our results clearly depicted that there **was** no significant relationship between radiological tumor size>2 cm and STAS with the combined OR of 1.47(95% CI:0.86–2.51; Fig. 2). The CT-based feature of radiologic tumor size larger than 2 cm cannot be used as a biomarker to predict STAS. This finding shows that tumors larger than 2 cm in CT image are not necessarily more likely to spread through air spaces in ADC.

### The association between pure solid nodule and STAS in ADC

**Fig. 3:**
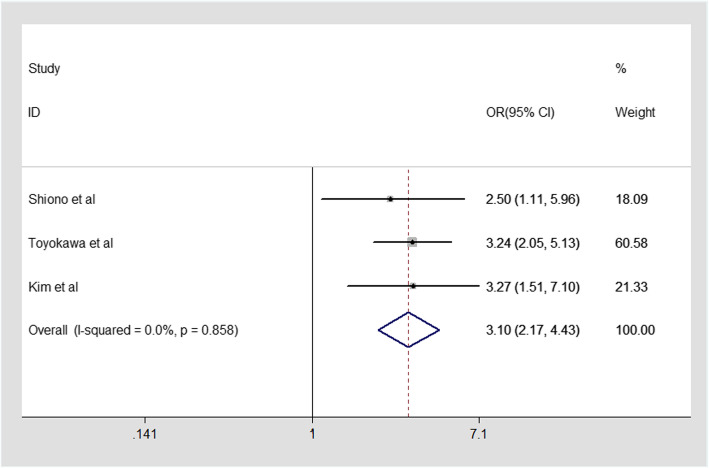
Meta-analysis of the association between pure solid nodule and STAS in ADC

In our mate-analysis, Three included studies evaluating the relationship between pure solid nodule in CT image and STAS were analyzed. Since there is no heterogeneity (I^2^ = 0.0%, *P* = 0.858),Fixed-effect model was applied. Our result revealed a strong association between pure solid nodule and STAS in ADC with pooled OR of 3.10(95%CI2.17–4.43, Fig. 3). Therefore, combined effect provided the evidence that pure solid nodule in CT image were more likely to spread through air spaces, which might be a marker to predict STAS in ADC.

### The association between part-solid nodule and STAS in ADC

**Fig. 4:**
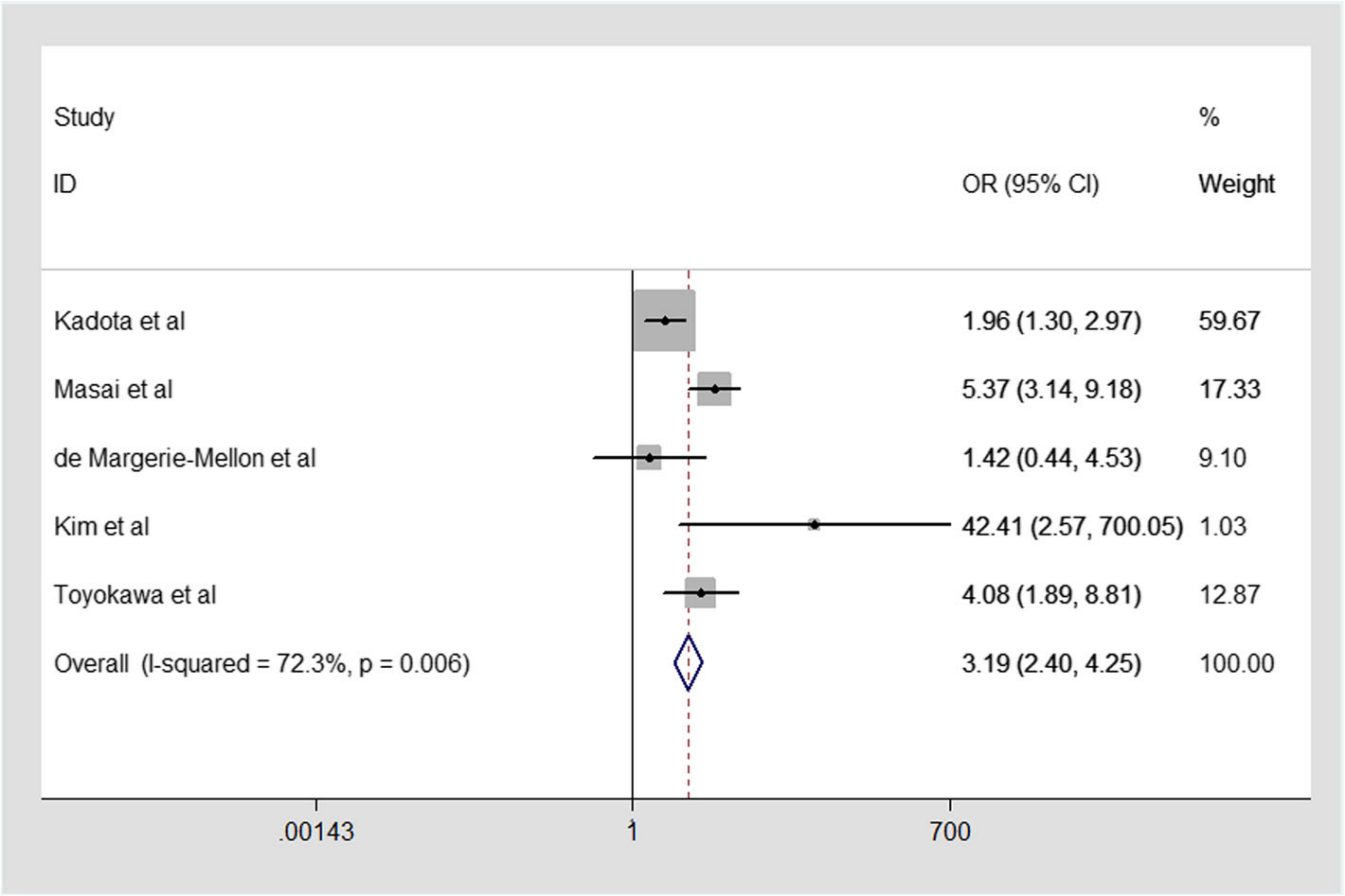
Meta-analysis of the association between part solid nodule and STAS in ADC

For the relationship between part-solid nodule in CT image and STAS in ADC. The five studies enrolled into our mate-analysis were analyzed by random effect model because of larger heterogeneity (I^2^ = 72.3%,*p* = 0.006, Fig. 4). From our mate-analysis, our result clearly showed that there **was** a significant association between part-solid nodule and STAS in ADC. Therefore, The part-solid nodule in CT scan may be more likely to appear STAS in ADC (combined OR:3.10,95%CI:2.17–4.43, Fig. 4).

### The association between percentage of solid component(PSC)>50% and STAS in ADC

**Fig. 5:**
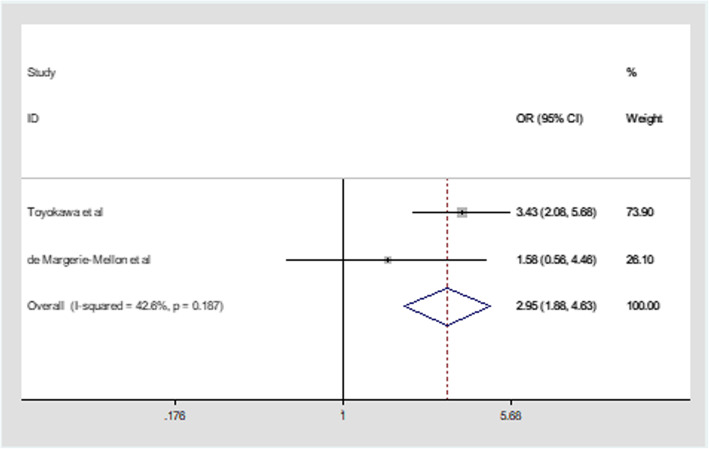
Meta-analysis of the association between PSC>50% and STAS in ADC

Percentage of the solid component (PSC) was defined as the ratio of maximum diameter of the solid component to the tumor average diameter multiplied by 100% in CT image. Two studies presented data to evaluated the association between PSC>50% and STAS in our mate-analysis. Fixed effect model was applied because of little heterogeneity (I^2^ = 42.6%,*p* = 0.187, Fig. 5). PSC>50% was a significant independent predictor in the diagnosis of STAS in ADC from our result with combined OR of 2.95(95%CI:1.88–4.63, Fig. 5). de Margerie-Mellon et al. [18] reported that high proportion of solid component diameter to tumor average diameter as CT manifestation in pulmonary adenocarcinomas was presented as a predictive biomarker in the diagnosis of STAS. From our mate-analysis, the tumor with PSC >50% is more likely to spread through air spaces in ADC.

### Sensitivity analysis and publication bias

**Fig. 6:**
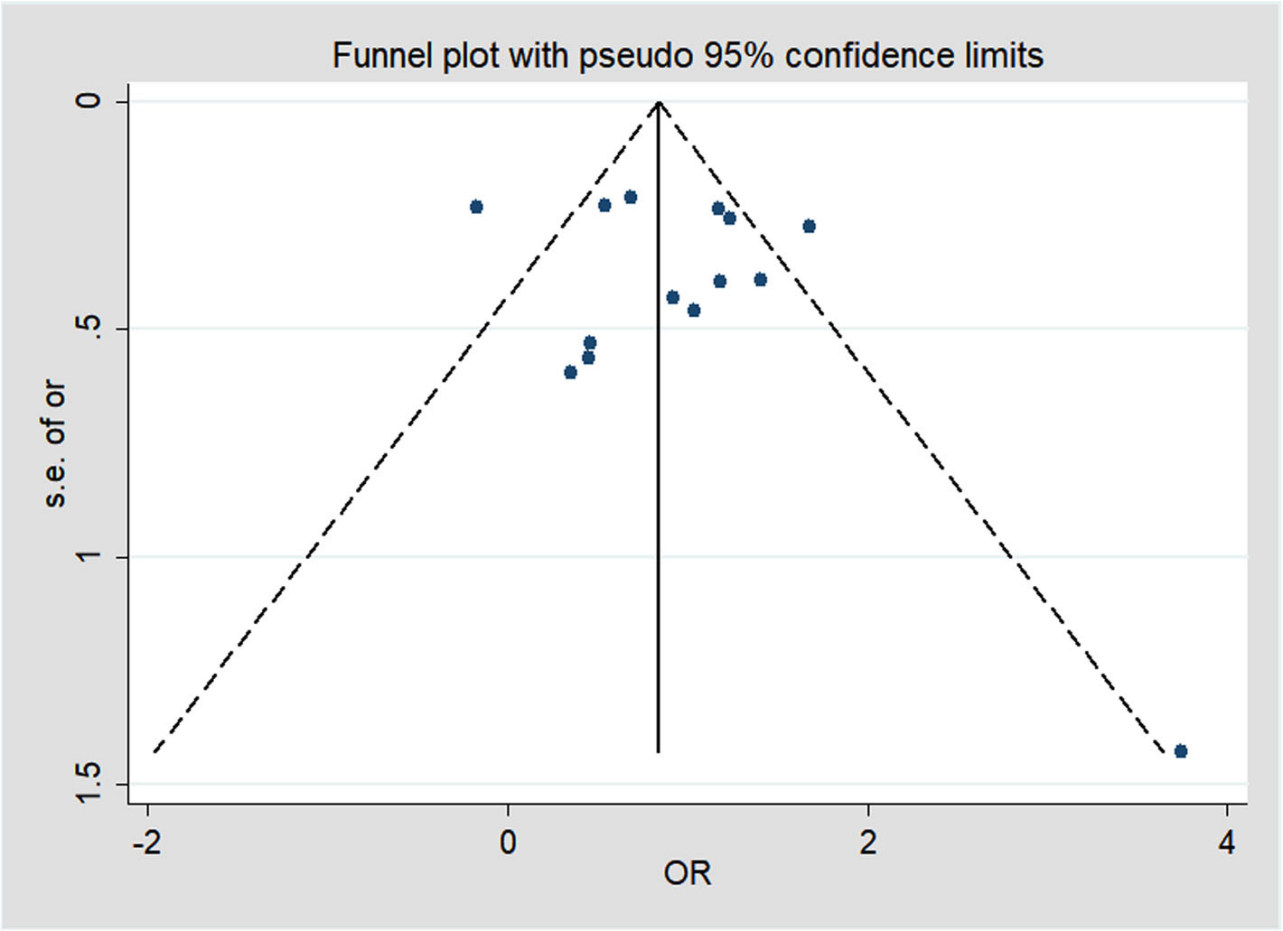
The funnel plot

The outcomes were similar whether fixed-effects models or random-effects models were used. Publication bias was evaluated by Begg’s funnel plot and Egger’s linear regression test, Egger’s test (*p* > 0.05) showed that there was no significant publication bias of studies included this meta-analysis. The shapes of the funnel plot are symmetric visually (Fig. 6) and no proof of publication bias was obtained.

## Discussions

To our knowledge. In recent years, there have been several studies exploring the relationship between preoperative CT findings and STAS of lung adenocarcinomas [17–19, 21]. de Margerie-Mellon and colleagues noticed that the size and rate of solid components were associated with STAS [18]. Kim et al. found that the percentage of solid component was an independent predictor of STAS [19]. Our primary result from this mate-analysis showed the CT-based features of pure solid nodule、part-solid nodule、PSC>50% have the potential to be a promising imaging **biomarkers** to preoperatively predict STAS, which could facilitate surgeons’ operation selection.

STAS is a recently described novel invasive pattern of lung cancer. Several studies showed that STAS was associated with recurrence and a decrease in overall survival in those patients with early-stage lung adenocarcinomas who received limited resection [4, 7, 10, 22]. However, if patients with STAS positive tumors had their entire lung lobe removed early, there was no significant association between STAS and tumor recurrence and overall patient survival [4]. Therefore, it is desirable for surgeons to choose the optimal operative type according to STAS status.

From our results, The CT characteristics of pure solid nodule、part-solid nodule、PSC>50% are promising imaging **biomarkers** for predicting STAS in ADC from our mate-analysis. STAS was more likely to manifest as pure solid nodule、part-solid nodule or PSC>50% in thin section CT image. de Margerie-Mellon et al. [18] reported that the nodules with maximum diameter of solid component≥1 cm were more likely to appear STAS with OR of 5.303(95%CI:1.85–15.23). Our conclusion is also consistent with current management guidelines and treatment strategies for resectable lung cancer. The National Comprehensive Cancer Network guidelines for non-small cell lung cancer suggest that sublobar resection is appropriate for small (≤2 cm) peripheral nodules with 50% or greater ground-glass appearance at CT [23]. In our study, the nodules with pure solid nodule、part-solid nodule or PSC>50%,which are more likely to spread through air spaces, are more suitable to choose standard lobectomy than sublobar resection in ADC. Toyokawa et al. [17] demonstrated that STAS-positive adenocarcinomas were significantly associated with a radiological tumor diameter larger than 2.0 cm (OR = 1.72,95CI%(1.11–2.70); *P* = 0.02). But our mate-analysis didn’t reach a same conclusion at this point. In our opinion, the majority of GGO with tumor diameter larger than 2.0 cm are unlikely to appear STAS in ADC. The other CT features, such as vascular convergence sign, vacuole sign, pleural indentation, spiculation, air bronchogram, haven’t been enrolled into our meta-analysis, because so little study has been done about them. Hopefully, more and more research will be done to evaluate the association between the other CT features and STAS in the future.

There are several limitations in our meta-analysis. First, the enrolled studies were retrospective, which contributed to that some bias were inevitable. Second, our meta-analysis only enrolled studies published in English language so as to some relevant studies in other languages were omitted, which might lead to publication bias and the limitation of applicable populations. Third, the number of enrolled studies is too low, in future, more studies with well-designed and large-scale are needed to confirm the conclusion. Fourth, our meta-analysis just provided evidence of association between CT-based features and STAS in ADC patients, but that doesn’t mean a causal relationship between them, cautious interpretation of the result is vital and more precise prospective studies is desperately needed.

## Conclusions

In conclusion, The CT-based features of pure solid nodule、part-solid nodule、PSC>50% are promising imaging **biomarkers** for predicting STAS in ADC and may substantially influence the choice of surgical type. In future, more studies with well-designed and large-scale are needed to confirm the conclusion.

## Data Availability

The datasets used and analysed during the current study are available from the corresponding author on reasonable request.

## Abbreviations

STAS: Spread through air spaces
GGO: Ground glass opacity
ADC: Adenocarcinoma
OR: Odd ratio
CI: Confidence interval
PSC: Percentage of solid component
NSCLC: Non-small cell lung cancer
HRCT: High resolution computed tomography
LDCT: Low-dose helical computed tomography
